# The Role of Quadruple Bonding in the Electron Transport through a Dimolybdenum Tetraacetate Molecule

**DOI:** 10.3390/molecules27206912

**Published:** 2022-10-14

**Authors:** Dmitry O. Arentov, Maxim R. Ryzhikov, Svetlana G. Kozlova

**Affiliations:** Nikolaev Institute of Inorganic Chemistry SB RAS, Acad. Lavrentiev Ave. 3, 630090 Novosibirsk, Russia

**Keywords:** DFT, NEGF, electron transport, Mo_2_(O_2_CCH_3_)_4_, d-orbitals

## Abstract

A dimolybdenum tetraacetate (Mo_2_(O_2_CCH_3_)_4_) molecule is embedded between two electrodes formed by semi-infinite 1D monatomic chains of lithium, aluminum, and titanium atoms. Electron transport through the Mo_2_(O_2_CCH_3_)_4_ molecule is calculated. The role of quadrupole bonding in the transport properties of the studied systems is analyzed.

## 1. Introduction

Dimolybdenum tetraacetate Mo_2_(O_2_CCH_3_)_4_ (DMT) is a molecule whose electronic structure is characterized by a quadruple Mo-Mo bonding with the σ^2^π^4^δ^2^ configuration where eight electrons are 4d valence electrons of two molybdenum atoms [1,2,3,4,5]. Theoretical chemistry pays particular attention to the properties of δ bonding, which is weaker than σ and π bonds but nonetheless affects the electronic absorption spectra and the spatial structure of the molecules with quadruple bonds [6,7]. Since DMT molecules can form ordered structures on various surfaces, they are currently considered as promising molecular units for the design of nanoscale electronic devices [8,9,10]. The role of σ, π, and δ bonds in the electron transport through a single molecule can also be discussed, since these bonds are formed by different overlaps of d-orbital lobes.

Electron transport through a single molecule is commonly described using a method based on the nonequilibrium Green’s function (NEGF) formalism [11,12,13]. The NEGF technique allows calculating the transmission function T(E) showing the sum probability for an electron with energy E to pass from the source to the drain through channels represented by the molecular orbitals (MOs) of the electrode—molecule—electrode system. T(E) depends both on the electronic structure of the electrodes and the molecule and on the interactions between them. In the case of weak contacts between the molecule and the electrodes, T(E) reflects the structure of energy levels of the MOs of the isolated molecule, and its shape is represented by a series of peaks (resonances) in the vicinity of MO energies [14]. Therefore, we assume that varying the distance between the contacts and the molecule can reveal T(E) peaks corresponding to individual MOs of the embedded molecule that can be used to analyze the participation of σ, π, δ bonds in the electron transport.

**Figure 1:**
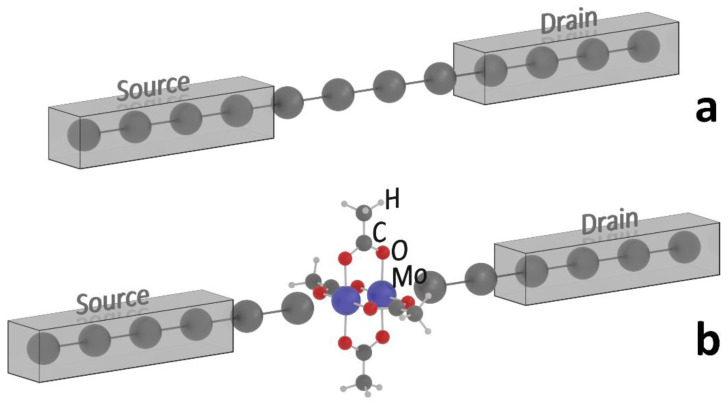
Model systems M_6_–M_6_ (**a**) and M_6_–DMT–M_6_ (**b**) (M = Li, Al, Ti).

In this work, we consider electron transport through a DMT molecule supplemented by two M atoms on both sides and embedded into a system of semi-infinite electrodes. The electrodes were represented by 1D chains of four M atoms (M = Li, Al, Ti) in the left (source) and right (drain) parts of the system, respectively. These s-, p-, and d-block metals are convenient models to study Fermi level shifts (E_F_). The 1D chain is the simplest model of electron container needed to calculate transmission function in a molecular system. The central part of the system was represented in two ways (Figure 1): by four M atoms for monoatomic wires (M_6_–M_6_) and by a DMT molecule embedded between M atoms of the monoatomic wires (M_6_–DMT–M_6_). System M_6_–M_6_ was used as a reference when studying transmission functions in systems (M_6_–DMT–M_6_).

## 2. Results and Discussion

### 2.1. Quadruple Bond in Mo_2_(O_2_CCH_3_)_4_ Molecule

**Figure 2:**
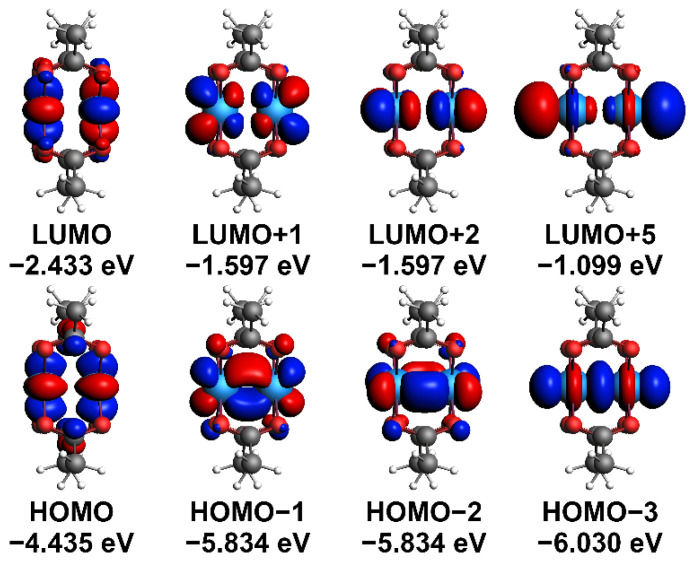
MOs (isosurface: 0.05 au) demonstrating bonding (σ, π, δ) and antibonding (σ*, π*, δ*) interactions of Mo 4d orbitals for the optimized DMT structure. The digits in parentheses are the MO energies in eV.

The optimized interatomic distances of the DMT molecule are Mo-Mo = 2.134 Å, Mo-O = 2.111 Å, and ∠Mo-Mo-O = 91.62° (see also Tables S1 and S2). These parameters agree with the corresponding experimental values Mo-Mo = 2.093 Å, <Mo-O> = 2.119 Å, and <∠Mo-Mo-O> = 91.8° [1]. Figure 2 shows MOs characterized by bonding (σ, π, δ) and antibonding (σ*, π*, δ*) interactions of Mo 4d orbitals, in agreement with the calculation results reported previously for the electronic structure of the DMT molecule [4]. Therefore, we assume that these MOs can be used to estimate the role of σ/σ*, π/π* and δ/δ* interactions in the electron transport through the molecule. The calculated HOMO—LUMO gap is 2.002 eV, in good agreement with the experimental value ~2.69 eV [7].

### 2.2. Electron Transport through Systems M_6_–(Mo_2_(O_2_CCH_3_)_4_)–M_6_ (M = Li, Al, Ti)

The M-Mo distances were varied from the sums of their empirical atomic radii (d(Li-Mo) = 2.90 Å, d(Al-Mo) = 2.70 Å, d(Ti-Mo) = 2.85 Å [15]) to 6–7 Å with 0.05 Å step. It was established that the peaks of transmission functions (T_DMT_(E)) of the M_6_–DMT–M_6_ system change their shape and energy positions. The peaks of T_DMT_(E) vanish above 6–7 Å. The T_DMT_(E) at the most indicative distances (the shortest, the distance where the peaks narrowing sharply, the longest, and points between them) are given at SI (Figures S1–S3).

#### 2.2.1. System Li_6_–(Mo_2_(O_2_CCH_3_)_4_)–Li_6_

**Figure 3:**
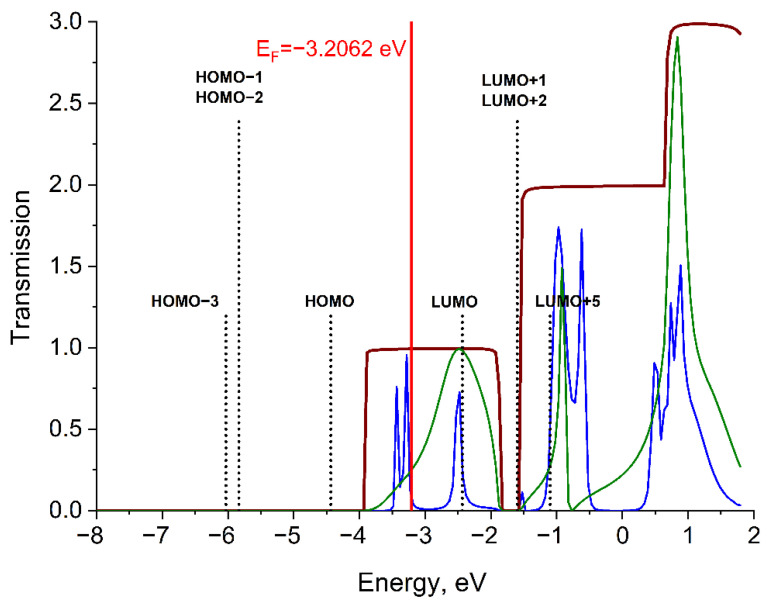
Transmission functions T_DMT_(E) (green for d(Li-Mo) = 2.90 Å and blue for d(Li-Mo) = 3.10 Å) for the Li_6_–DMT–Li_6_ system and transmission function T_wire_(E) (brown) for the Li_6_-Li_6_ system. E_F_ denotes the Fermi level of electrodes. Energy positions of bonding (σ, π, δ) and antibonding (σ*, π*, δ*) MOs are shown by vertical lines (black dots).

Function T_wire_(E) for the Li wire has three plateaus (Figure 3). The embedding of a DMT molecule into a wire of lithium atoms decreases the T_DMT_(E) values compared to the T_wire_(E) values. The T_DMT_(E) for d(Li-Mo) = 2.90 Å has three humps in the energy range where function T_wire_(E) for the Li wire has three plateaus. The peaks of these humps are located at −2.45 eV, −0.92 eV, and 0.84 eV energies. Function T_DMT_(E) begins narrowing sharply at the d(Li-Mo) distance equal to 3.10 Å (Figure S1). The T_DMT_(E) function for d(Li-Mo) = 3.10 Å has peaks in the same regions at −3.43 eV, −3.28 eV, −2.48 eV, −0.97 eV, and −0.62 eV as well as some peaks above 0 eV.

The LUMO and LUMO+5 (corresponding to δ* and σ* interactions, respectively) of the DMT molecule are the only orbitals that fall into non-zero value regions of both functions T_wire_(E) and T_DMT_(E). On the one side, the LUMO+1 and LUMO+2 are close to the dip (−2; −1.5 eV) of function T_wire_(E). On the other, these two orbitals are close to the T_DMT_(−1.5eV) peak at d(Li-Mo) = 3.1 Å. Thus, the participation of LUMO+1 and LUMO+2 (corresponding to two π* interactions) in electron transport is ambiguous. The HOMO, HOMO-1, HOMO-2, and HOMO-3 are located beyond the transmission bands and apparently do not participate in conduction. As can be seen, the 1D chain can’t be considered as a simple bulk electron container, since the transmission function of unperturbed by molecule 1D chain have some structure with humps, dips and plateau [16]. Thus, the DMT can provide channels for electrons only in the region where the transmission of the reference 1D chain of Li is non-zero.

#### 2.2.2. System Al_6_–(Mo_2_(O_2_CCH_3_)_4_)–Al_6_

**Figure 4:**
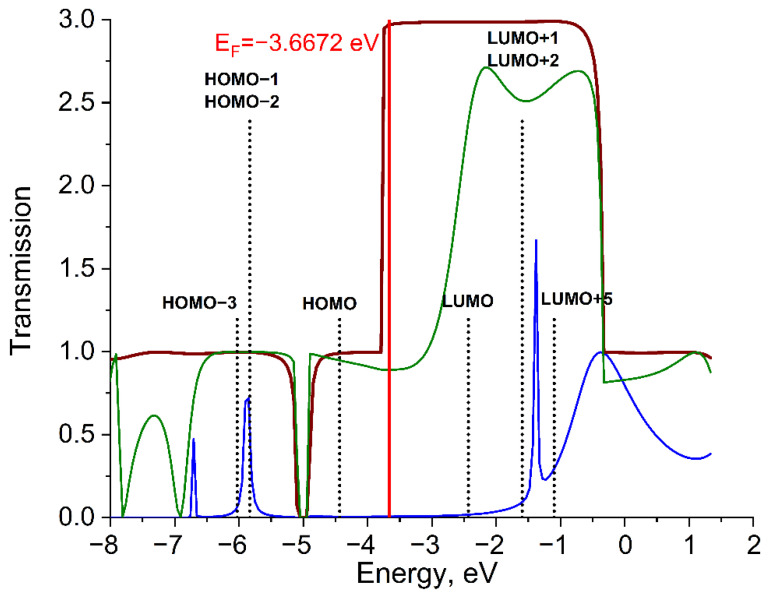
Transmission functions T_DMT_(E) (green for d(Al-Mo) = 2.70 Å and blue for d(Al-Mo) = 4.30 Å) for the Al_6_–DMT–Al_6_ system and transmission function T_wire_(E) (brown) for the Al_6_-Al_6_ system. E_F_ denotes the Fermi level of electrodes. Energy positions of bonding (σ, π, δ) and antibonding (σ*, π*, δ*) MOs are shown by vertical lines (black dots).

The T_DMT_(E) function of the Al_6_–DMT–Al_6_ system for d(Al-Mo) = 2.70 Å has close to unity values over the largest part of the calculated energy range (Figure 4). The only exception is the energy range from −2.7 eV to −0.27 eV, where T_DMT_(E) is close to 3. Function T_DMT_(E) deviates from T_wire_(E) in the energy region from −7.8 eV to −6.9 eV and above the Fermi level (−3.7 eV) up to 0.3 eV (Figure 4). The transmission function became more resolved at d(Al-Mo) = 4.30 Å, still keeping most of features of short distance T_DMT_(E). Function T_DMT_(E) for d(Al-Mo) = 4.30 Å is represented by peaks at −6.69 eV, −5.87 eV, −1.38 eV, and −0.36 eV. The DMT molecule suppresses electron transport compared to the reference Al wire, similar to the system with Li. 

The energy regions of functions T_wire_(E) and T_DMT_(E) (for d(Al-Mo) = 2.70 Å) contain DMT MOs exhibiting bonding (σ, π, δ) and antibonding (σ*, π*, δ*) interactions. At d(Al-Mo) = 4.30 Å, HOMO-3 (σ-bond), HOMO-2 and HOMO-1 (two π-bonds), LUMO+1 and LUMO+2 (two π*-interactions), LUMO+5 (σ*-interaction) can contribute to T_DMT_(E). The δ/δ*-interactions (HOMO and LUMO) don’t participate in transmission at this distance.

#### 2.2.3. System Ti_6_–(Mo_2_(O_2_CCH_3_)_4_)–Ti_6_

**Figure 5:**
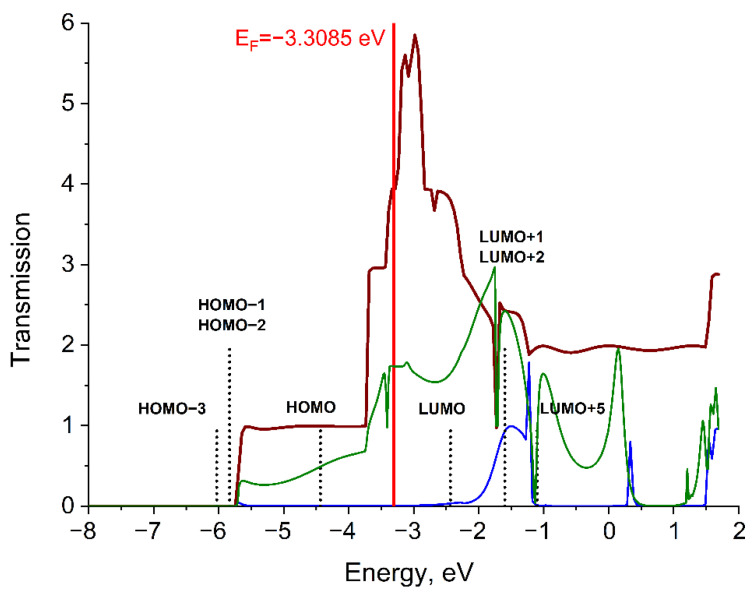
Transmission functions T_DMT_(E) (green for d(Ti-Mo) = 2.85 Å and blue for d(Ti-Mo) = 4.80 Å) for the Ti_6_–DMT–Ti_6_ system and transmission function T_wire_(E) (brown) for the Ti_6_-Ti_6_ system. E_F_ denotes the Fermi level of electrodes. Energy positions of bonding (σ, π, δ) and antibonding (σ*, π*, δ*) MOs are shown by vertical lines (black dots).

Function T_DMT_(E) is smaller than T_wire_(E) over the largest part of the calculated energy range, meaning that the embedded molecule significantly affects the electron transport through the Ti wire (Figure 5). The energy ranges of functions T_wire_(E) and T_DMT_(E) (for d(Ti-Mo) = 2.85 Å) contain HOMO, LUMO, LUMO+1, LUMO+2, and LUMO+5. For d(Ti-Mo) = 4.80 Å, the region of non-negligible T_DMT_(E) values contains only LUMO+1 and LUMO+2 (two π*-interactions).

#### 2.2.4. Discussion 

The Mo-Mo distance in DMT (2.13 Å) is smaller than interatomic distances in 1D chains (2.86 Å–3.04 Å). The DMT molecule can be viewed as a defect disturbing the electronic structure of the monatomic wire. In addition, the embedded DMT molecule can be considered as a kind of filter that blocks some of the electron transport channels originally present in the 1D monatomic chains. This idea can explain the fact that the transmission functions of systems with an embedded DMT molecule are smaller than those of the wires and that not all MOs exhibiting bonding (σ, π, δ) and antibonding (σ*, π*, δ*) interactions of Mo 4d orbitals can participate in the electron transport through the DMT molecule.

## 3. Theoretical Calculations

The geometry of the dimolybdenum tetraacetate (DMT) molecule was optimized by the ADF engine of the AMS2020 package [17,18] using the BP86 density functional [19] and the TZP all-electron basis set for all atoms [20]. All of the calculated vibrational frequencies were real, indicating that the optimized structure of the DMT molecule corresponds to the energy minimum (Table S1). Scalar relativistic effects were considered within the ZORA approach [21].

The electron transport was studied using the nonequilibrium Green’s function (NEGF) formalism [11,12,13] as implemented in the BAND engine of the AMS2020 package [22,23], the BP86 density functional [24,25,26], and the ZORA [27] approach for scalar relativistic effects.

To reproduce the electronic properties of bulk metals most closely, the interatomic distances in lithium (d(Li-Li) = 3.040 Å), aluminum (d(Al-Al) = 2.864 Å), and titanium (d(Ti-Ti) = 2.951 Å) chains were chosen equal to the interatomic distances in the corresponding crystals [28,29,30]. The atoms of the studied systems were calculated using the TZP basis set [18] with the following frozen cores: Li(1s), Al(1s.2p), Ti(1s.2s.2p.3s.3p), Mo(1s.2s.2p.3s.3p.3d.4s.4p), O(1s), C(1s).

The GGA-BP86 density functional was chosen due to its time cost advantages in T(E) calculations [31]. For efficiency reasons, the radial part of the basis set functions is multiplied by a Fermi—Dirac function controlled by two main parameters: distance from the center R = 7 Å and decay width Δ = 0.7 [23]. Function T(E) was calculated using the self-consistent field method in the energy range −5 eV < E − E_F_ < 5 eV at a temperature of 316 K (corresponding to the kT energy of 0.001 Hartree).

## 4. Conclusions

It was shown on the example of M_6_–DMT–M_6_ systems (M = Li, Al, Ti) that an embedded DMT molecule can be considered as a defect decreasing the transmission function. It was revealed that the transmission function depends on d(Mo-M) distances. It was shown that increasing the d(Mo-M) values affects not only the shape of the transmission function but also the number of DMT MOs involved in the electron transport through the molecule. No dependence of the transmission function on different overlaps of d-orbital lobes of Mo was revealed. For M_6_–DMT–M_6_ (M = Al, Ti) systems, the highest values of transmission functions were found in the region of LUMO+1 and LUMO+2 characterized by the π* interaction. However, these values can be explained not by the overlaps of d-orbital lobes but by the presence of two such MOs.

Although the results of this work may seem purely theoretical, they provide a first insight into the role of overlapping d-orbital lobes in the electron transport through a molecule. The relevance of the present study is substantiated by the success that has been achieved in experimental works devoted to the fabrication of 1D monatomic chains of metals [32]. The greatest progress in this field was achieved for gold wires [33]. For many other metals, nanowires with a diameter smaller than 20 nm were obtained, and the work continues to reduce their thickness still further [34]. In this regard, the transition to monatomic wires can be viewed as the next step towards the design of single molecule devices.

## Data Availability

The data presented in this study are available on request from the corresponding author.

## Supplementary Materials

The following supporting information can be downloaded at: https://www.mdpi.com/article/10.3390/molecules27206912/s1, Figure S1: Transmission functions in systems Li_6_–(Mo_2_(O_2_CCH_3_)_4_)–Li_6_ depending on distances d(Li-Mo); Figure S2: Transmission functions in systems Al_6_–(Mo_2_(O_2_CCH_3_)_4_)–Al_6_ depending on distances d(Al-Mo). Figure S3: Transmission functions in systems Ti_6_–(Mo_2_(O_2_CCH_3_)_4_)–Ti_6_ depending on distances d(Ti-Mo). Table S1: Optimized coordinates (Å) of Mo_2_(O_2_CCH_3_)_4_ molecule (BP86/TZP level); Table S2: Frequencies and their intensities of Mo_2_(O_2_CCH_3_)_4_ molecule.
