# Supplementary material for: The Role of Quadruple Bonding in the Electron Transport through a Dimolybdenum Tetraacetate Molecule
Source: Molecules. 2022 Oct 14;27(20):6912. doi: 10.3390/molecules27206912 (PMC9609368; doi:10.3390/molecules27206912)
Supplement: Supplementary file 1 [file molecules-27-06912-s001.zip › molecules-1972538-supplementary.pdf]

Supplementary to

The role of quadruple bonding in the electron transport through a dimolybdenum tetraacetate molecule

Dmitry O. Arentov, Maxim R. Ryzhikov, Svetlana G. Kozlova

Nikolaev Institute of Inorganic Chemistry SB RAS, Acad. Lavrentiev Ave. 3, Novosibirsk, Russia, 630090

I. Optimized coordinates of $Mo_2(O_2CCH_3)_4$ molecule

Table S1. Optimized coordinates (Å) of $Mo_2(O_2CCH_3)_4$ molecule (BP86/TZP level)

Mo	0.018493000	1.263767000	-1.369294000
Mo	0.143704000	1.259600000	0.761234000
O	-1.482136000	-0.220839000	-1.343649000
O	-1.467431000	2.764109000	-1.339183000
O	1.511717000	2.748140000	-1.513947000
O	1.496465000	-0.236806000	-1.519547000
O	1.645410000	2.744495000	0.736029000
O	-1.350191000	-0.225996000	0.906426000
O	1.628627000	-0.240882000	0.730503000
O	-1.334075000	2.759085000	0.910803000
C	-1.858637000	-0.662670000	-0.193592000
C	-1.840088000	3.203921000	-0.187307000
C	2.022471000	3.184131000	-0.414525000
C	2.000167000	-0.682859000	-0.421151000
C	-2.906317000	4.262576000	-0.111241000
C	3.056322000	-1.753042000	-0.474410000
C	3.099409000	4.232107000	-0.487168000
C	-2.927207000	-1.720602000	-0.143985000
H	-2.560881000	5.095705000	0.515179000
H	-3.803294000	3.845488000	0.368952000
H	-3.163362000	4.623309000	-1.111868000
H	-3.141647000	-2.003875000	0.890893000
H	-2.601241000	-2.602495000	-0.712734000
H	-3.841409000	-1.345462000	-0.624847000
H	3.360133000	4.586813000	0.514656000
H	2.762474000	5.070835000	-1.110757000
H	3.992083000	3.807560000	-0.968847000
H	3.972629000	-1.393033000	0.013847000
H	2.716844000	-2.635524000	0.085517000
H	3.272019000	-2.030821000	-1.510526000

II. Normal mode frequencies of $Mo_2(O_2CCH_3)_4$ molecule

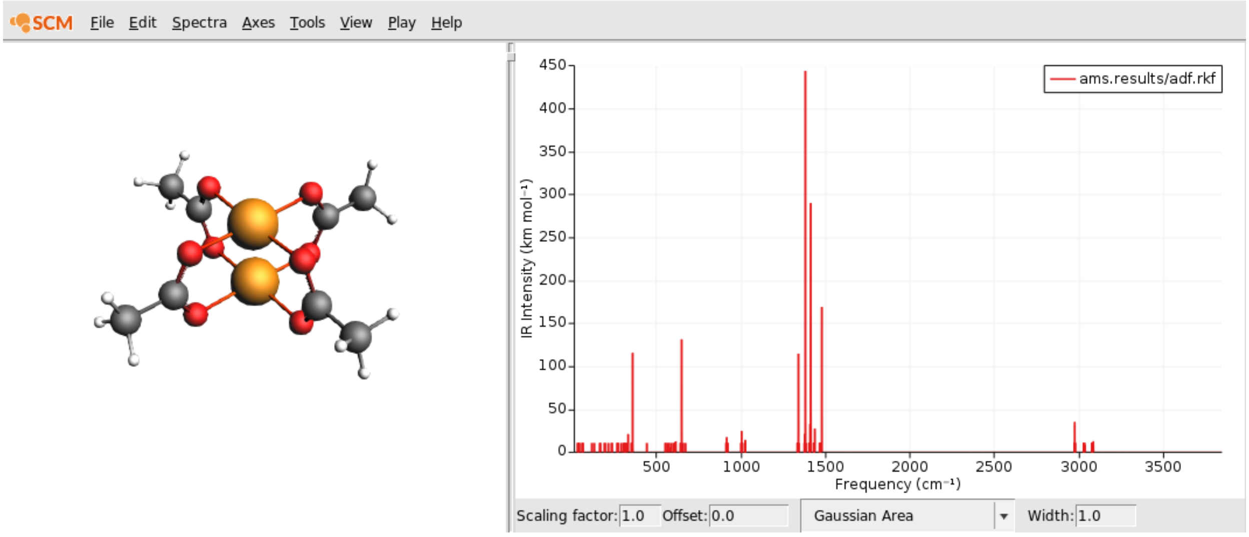

Table S2. Frequencies and their intensities of $Mo_2(O_2CCH_3)_4$ molecule

Index	Frequency (cm-1)	Intensity (km/mol)	Index	Frequency (cm-1)	Intensity (km/mol)
7	34.8774	0.6898	49	916.0544	0.0387
8	35.3763	0.5256	50	919.8345	0.0023
9	37.0806	0.2237	51	998.8525	7.4516
10	38.4586	0.2130	52	998.9173	8.2355
11	57.6190	0.2541	53	999.2003	13.5917
12	57.7435	0.2454	54	999.5575	0.0173
13	61.0791	0.0001	55	1019.8722	0.5422
14	116.6129	0.0000	56	1020.3140	0.1938
15	129.2829	0.0014	57	1020.9176	8.6713
16	163.8845	0.0003	58	1020.9618	6.6006
17	164.7466	1.6498	59	1333.5797	0.9209
18	194.9430	0.0238	60	1335.4134	63.4634
19	195.2910	0.0220	61	1335.5508	61.9005
20	209.8812	0.0019	62	1339.5928	0.0056
21	230.8103	1.0945	63	1373.1658	22.7134
22	231.2867	1.0355	64	1376.7386	227.8776
23	266.2761	0.0001	65	1376.7946	246.5968
24	268.1887	0.0020	66	1383.9744	0.0022
25	268.2558	0.0015	67	1404.6370	23.5693
26	289.7828	0.0006	68	1405.0387	15.5889
27	300.9177	0.0168	69	1406.2123	2.2614
28	308.3408	0.0057	70	1408.9892	308.6701
29	308.6801	0.0070	71	1432.0867	3.4350
30	315.6764	0.0002	72	1432.3846	9.9068
31	326.8429	22.5936	73	1432.6534	18.4645
32	353.4987	68.0910	74	1432.7480	1.6351
33	353.8923	69.0509	75	1466.9406	2.4405
34	439.4197	0.0000	76	1467.3579	2.5200
35	552.2767	0.2943	77	1467.6615	0.3084
36	552.3691	0.2707	78	1474.0395	179.9358
37	563.7880	0.0003	79	2972.2868	13.0427
38	576.2474	5.6697	80	2972.3899	12.5662
39	590.2648	0.1211	81	2972.7480	11.6201
40	602.7719	0.1071	82	2972.8543	7.4407
41	608.9827	12.0338	83	3032.8885	3.9399
42	609.6997	6.7552	84	3032.9034	3.9491
43	643.7782	90.9040	85	3033.8575	3.3542
44	644.2213	84.3721	86	3033.9335	5.1245
45	653.9598	0.0231	87	3082.0098	4.3431
46	670.1253	0.0103	88	3082.0275	3.3638
47	911.5106	9.0473	89	3082.4673	2.2001
48	911.5579	9.6245	90	3082.5457	7.2808

III. Transmission functions in systems M_6–($Mo_2(O_2CCH_3)_4$)–M_6 depending on distances d(M-Mo), M=Li, Al and Ti

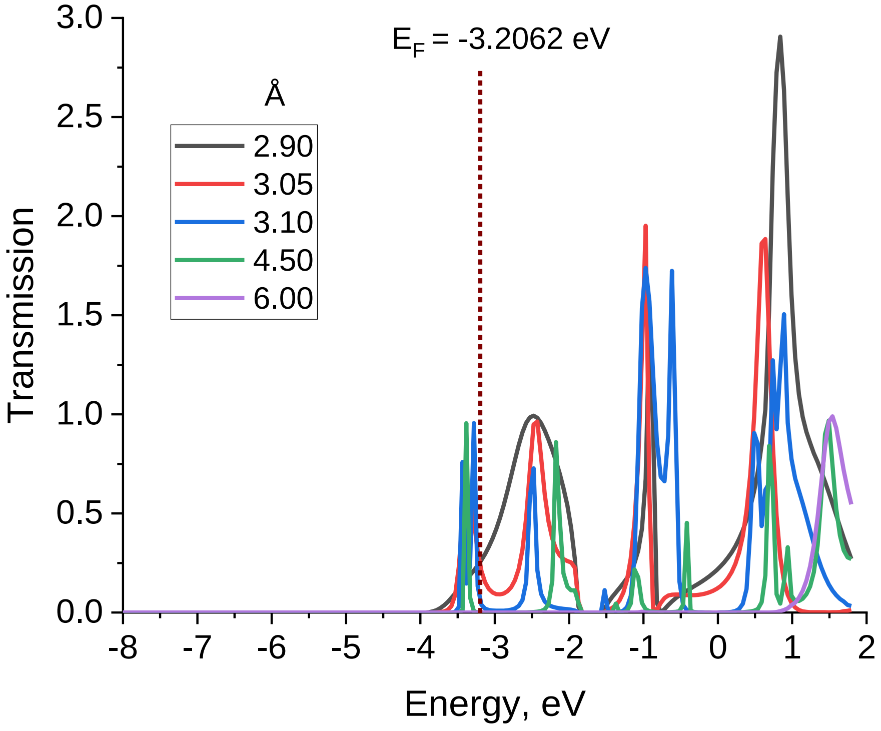

Figure S1. Transmission functions in systems Li_6–($Mo_2(O_2CCH_3)_4$)–Li_6 depending on distances d(Li-Mo).

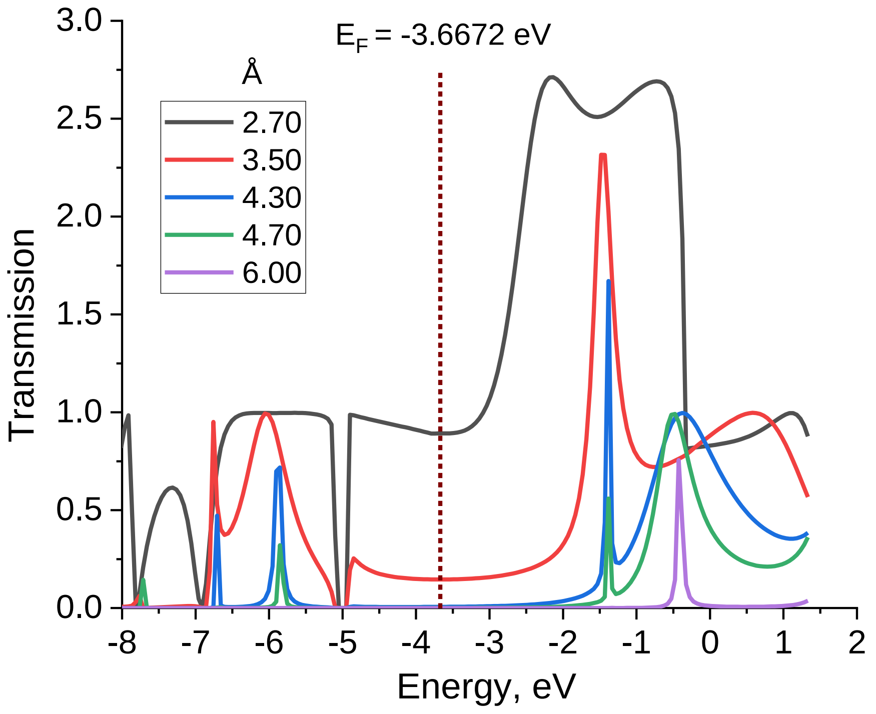

Figure S2. Transmission functions in systems Al_6–($Mo_2(O_2CCH_3)_4$)–Al_6 depending on distances d(Al-Mo).

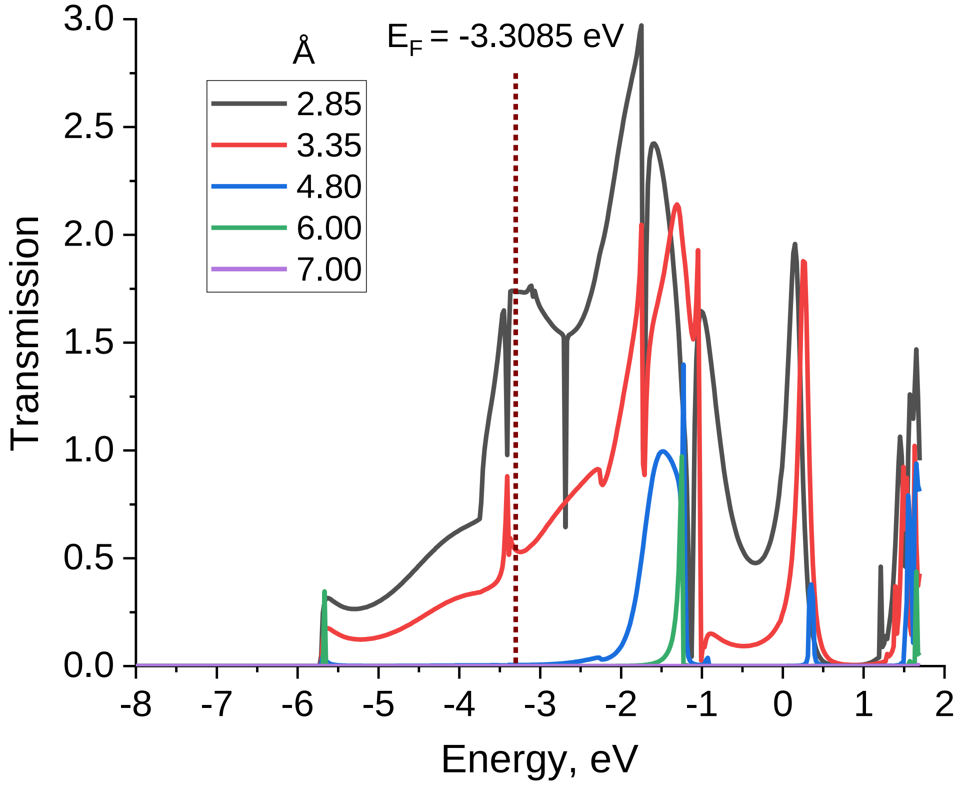

Figure S3. Transmission functions in systems Ti_6–($Mo_2(O_2CCH_3)_4$)–Ti_6 depending on distances d(Ti-Mo).
